# Efficacy of a mouthwash containing ε-poly-L-lysine, funme peptides and domiphen in reducing halitosis and supragingival plaque: a randomized clinical trial

**DOI:** 10.1186/s12903-024-04255-0

**Published:** 2024-05-03

**Authors:** Song Shen, Xu Liu, Jun Huang, Yi Sun, Bin Liu, Wenzhu Song, Lei Meng, Mi Du, Qiang Feng

**Affiliations:** 1 Department of Human Microbiome & Implantology, School and Hospital of Stomatology, Cheeloo College of Medicine, Shandong Key Laboratory of Oral Tissue Regeneration & Shandong Engineering Laboratory for Dental Materials and Oral Tissue Regeneration & Shandong Provincial Clinical Research Center for Oral Diseases, Shandong University, Jinan, China; 2 Shandong University-BOP Joint Oral Microbiome Laboratory, Shandong University, Jinan, 250012 China; 3 Shanghai Gemang Bio-Technology Co., Ltd, Shanghai, China

**Keywords:** Mouthwash, Antimicrobial peptides, Minimum inhibitory concentration, Minimum bactericidal concentration, Supragingival plaque, Halitosis

## Abstract

**Objective:**

To evaluate the antibacterial effectiveness of a combination of ε-poly-L-lysine (ε-PL), funme peptide (FP) as well as domiphen against oral pathogens, and assess the efficacy of a BOP® mouthwash supplemented with this combination in reducing halitosis and supragingival plaque in a clinical trial.

**Materials and methods:**

The minimum inhibitory concentration (MIC) and minimum bactericidal concentration (MBC) of the compound against *Fusobacterium nucleatum*, *Porphyromonas gingivalis*, *Streptococcus mutans*, and *Aggregatibacter actinomycetemcomitans* were determined by the gradient dilution method. Subsequently, the CCK-8 assay was used to detect the toxicity of mouthwash on human gingival fibroblastst, and the effectiveness in reducing halitosis and supragingival plaque of the mouthwash supplemented with the combination was analyzed by a randomized, double-blind, parallel-controlled clinical trial.

**Results:**

The combination exhibited significant inhibitory effects on tested oral pathogens with the MIC < 1.56% (v/v) and the MBC < 3.13% (v/v), and the mouthwash containing this combination did not inhibit the viability of human gingival fibroblasts at the test concentrations. The clinical trial showed that the test group displayed notably lower volatile sulfur compounds (VSCs) at 0, 10, 24 h, and 7 d post-mouthwash (*P* < 0.05), compared with the baseline. After 7 days, the VSC levels of the and control groups were reduced by 50.27% and 32.12%, respectively, and notably cutting severe halitosis by 57.03% in the test group. Additionally, the Plaque Index (PLI) of the test and control group decreased by 54.55% and 8.38%, respectively, and there was a significant difference in PLI between the two groups after 7 days (*P* < 0.01).

**Conclusions:**

The combination of ε-PL, FP and domiphen demonstrated potent inhibitory and bactericidal effects against the tested oral pathogens, and the newly formulated mouthwash added with the combination exhibited anti-dental plaque and anti-halitosis properties in a clinical trial and was safe.

**Trial registration:**

The randomized controlled clinical trial was registered on Chinese Clinical Trial Registry (No. ChiCTR2300073816, Date: 21/07/2023).

**Supplementary Information:**

The online version contains supplementary material available at 10.1186/s12903-024-04255-0.

## Background

The oral cavity constitutes a complex microbial ecosystem, ranking as the second-largest microbial community following the intestines [1]. Oral microbiota is a unique community of microorganisms (at least 10^8^ microbial cells/mL saliva) and over 700 species of microbes have been identified [2]. Bacteria are predominant microbes colonizing in oral cavity along with fungi, viruses, archaea, and protozoa [3]. These diverse oral microorganisms extensively colonize the surfaces of oral hard tissues (teeth) and mucosal tissues (lips, buccal mucosa, tongue, mouth floor, palate, gingival mucosa), forming microecological communities characterized by distinct species compositions and proportions [4]. These communities play a crucial role in maintaining the balance of the host oral ecosystem and promoting overall oral health [5].

Dental plaque represents a highly organized and intricate oral biofilm ecosystem complicatedly linked to various oral diseases [6]. This viscid biofilm comprises a consortium of diverse oral microorganisms embedded in an extracellular matrix, commonly adhering to both the tooth surface and the gingival margin [7]. Dental plaque biofilm is classified into two categories including supragingival and subgingival plaque [8, 9]. *Streptococcus mutans* (*S. mutan*s), *Lactobacillus*, and *Actinomyces* species residing in supragingival plaque are closely related to dental caries, whereas *Porphyromonas gingivalis* (*P. gingivalis*), *Fusobacterium nucleatum* (*F. nucleatum*), and *Treponema denticola* in subgingival plaque are closely related to periodontal disease [10]. Based on microbial-substrate interactions, some oral microbiota, especially periodontal pathogens can degrade or metabolize organic substrates to be volatile sulfur compounds (VSCs) causing halitosis (oral malodor) [11]. Therefore, maintaining the balance of oral microbiota is an essential and recommended strategy to oral health since the dysbiosis of such microbes leads to numerous oral and systemic diseases.

Mouthwash is an antiseptic liquid preparation frequently used to clean oral cavity and freshen the breath [12]. In order to reduce plaque and bad breath, mouthwash has been enriched with a number of antibacterial ingredients, such as chlorhexidine, esssetial oil and delmopinol. However, these ingredients come with many side effects, such as staining, discomfort and oral numbness [13], new antimicrobial agents need to be investigated and added to mouthwash to promote oral hygiene.

Domiphen, ε-poly-L-lysine (ε-PL), and funme peptide (FP) are three potent antibacterial agents of interest. Domiphen is a broad-spectrum antibacterial agent with cationic surfactant property by changing microbial membrane permeability and disrupting their metabolism [14]. ε-PL and FP are classified as antimicrobial peptides (AMPs), which are peptide molecules with broad-spectrum antibacterial, anti-inflammatory and immunomodulatory activities [15]. AMPs can actively inhibit oral pathogens with less propensity for development of antimicrobial resistance [16], and have been progressively included into the dental fields, such as antibacterial agents [17] and implant coatings [18]. ε-PL is a natural polypeptide consisting of 25–30 L-lysine units that is biodegradable, water-soluble, nontoxic, and edible [19, 20]. FP, a novel amphiphilic α-helical antimicrobial peptide composing of 19 amino acids, has been commercially used in skin and periodontal diseases as the core active ingredient of antibacterial products [21].

Consequently, a newly developed antimicrobial combination derived from ε-PL, FP and domiphen was studied in vitro for its inhibitory and bactericidal effects against some oral pathogens. The mouthwash supplemented with this newly developed antibacterial combination was also studied in vivo for its efficacy to reduce halitosis and supragingival plaque formation.

## Materials and methods

### Antibacterial and cytotoxicity tests

#### Main reagents

Heme chloride and vitamin K1 were purchased from Qingdao Hi-tech Industrial Park Hope Bio-technology Co., Ltd, sterile defidrinated sheep blood was purchased from Guangzhou Hongquan Biotechnology Co., Ltd. The stock solution of heme chloride was prepared according to the ratio of heme chloride: distilled water: sodium hydroxide = 1:5:40, filtered and sterilized, and stored at 4 ℃ in the dark.

Brain Heart Infusion (BHI) medium, purchased from Becton, Dickinson and Company. According to the instructions, the solid medium was added with 1.5–2% agar, autoclaved. When the solution was cooled to about 50 ℃, 5% sterile defiber sheep blood, 0.05% vitamin K1, and 0.1% hemin chloride stock solution were added, mixed, and divided into plates, sealed after cooling into solid, and stored in the dark at 4 ℃ for later use.

The combination of three antibacterial ingredients: ε-PL (150 µg/mL), FP (0.25 µg/mL) and domiphen (300 µg/mL).

Test strains: *F. nucleatum* ATCC 25,586, *P. gingivalis* ATCC 33,277, *S. mutans* NBRC 13,955, and *A. actinomycetemcomitans* HK 1651. All four strains were cultured using BHI medium, *F. nucleatum* and *P. gingivalis* were cultured under anaerobic conditions, and *S. mutans* and *A. actinomycetemcomitans* were cultured under aerobic conditions.

#### Determination of minimum inhibitory concentration (MIC) and minimum bactericidal concentration (MBC)

The two-fold broth micro dilution method was used to detect the MIC of the mixed antibacterial solution of the three ingredients against the four pathogens. At first, sterile 96-well culture plates were prepared, and the antibacterial solution was added in a final concentration gradient from 25% (v/v) to 0.10% (v/v) in 1st to 9th wells, and the chlorhexidine (0.5%) was added in 10th wells as a positive control, no drug in 11th wells as a negative control, and only medium in 12th wells as a blank control. Then, the bacterial broth in the logarithmic growth phase was diluted with BHI liquid medium and added to 96-well plates to achieve a final concentration of 1 × 10^8^ CFU/mL. Three parallel groups were prepared for each strain. Subsequently, the 96-well plates containing bacterial and antimicrobial solution were placed in a constant temperature incubator at 37 °C for 20 to 24 h. The turbidity of the medium in each well was visually assessed at the end of the incubation period, and the lowest drug concentration at which the medium remained clear was designated as the MIC for the antimicrobial solution.

The bacterial solution from the well exhibiting the MIC and its four adjacent above wells were individually transferred and inoculated on culture agar plate. Following 24 h of incubation in a 37 °C incubator, the MBC was the concentration of the tested drug that completely eliminated the tested bacteria by showing no colony appearance.

#### Cell viability detection using a cell counting kit 8 assay

The effect of the mouthwash on the viability of human gingival fibroblasts (HGFs) was evaluated by a CCK-8 cell counting kit (CCK-8; Biosharp, China). HGFs were seeded into 96-well plates with high-glucose DMEM medium (BasalMedia Technologies Co., Shanghai, China), and transferred to a CO_2_ incubator for 24 h at 37 °C. Cells were then stimulated with the different concentrations of test mouthwash for 30 s, and a mouthwash containing 0.12% chlorhexidine and 0.02% metronidazoleand as the positive control, only medium as the negative control. Afterwards, the cells were incubated in a medium containing 10 µL test reagents for 1 h at 37 ℃. The optimal absorbance at 450 nm was determined using a microplate reader.

### Clinical trials on halitosis and supragingival plaque reduction

#### General information

**Fig. 1:**
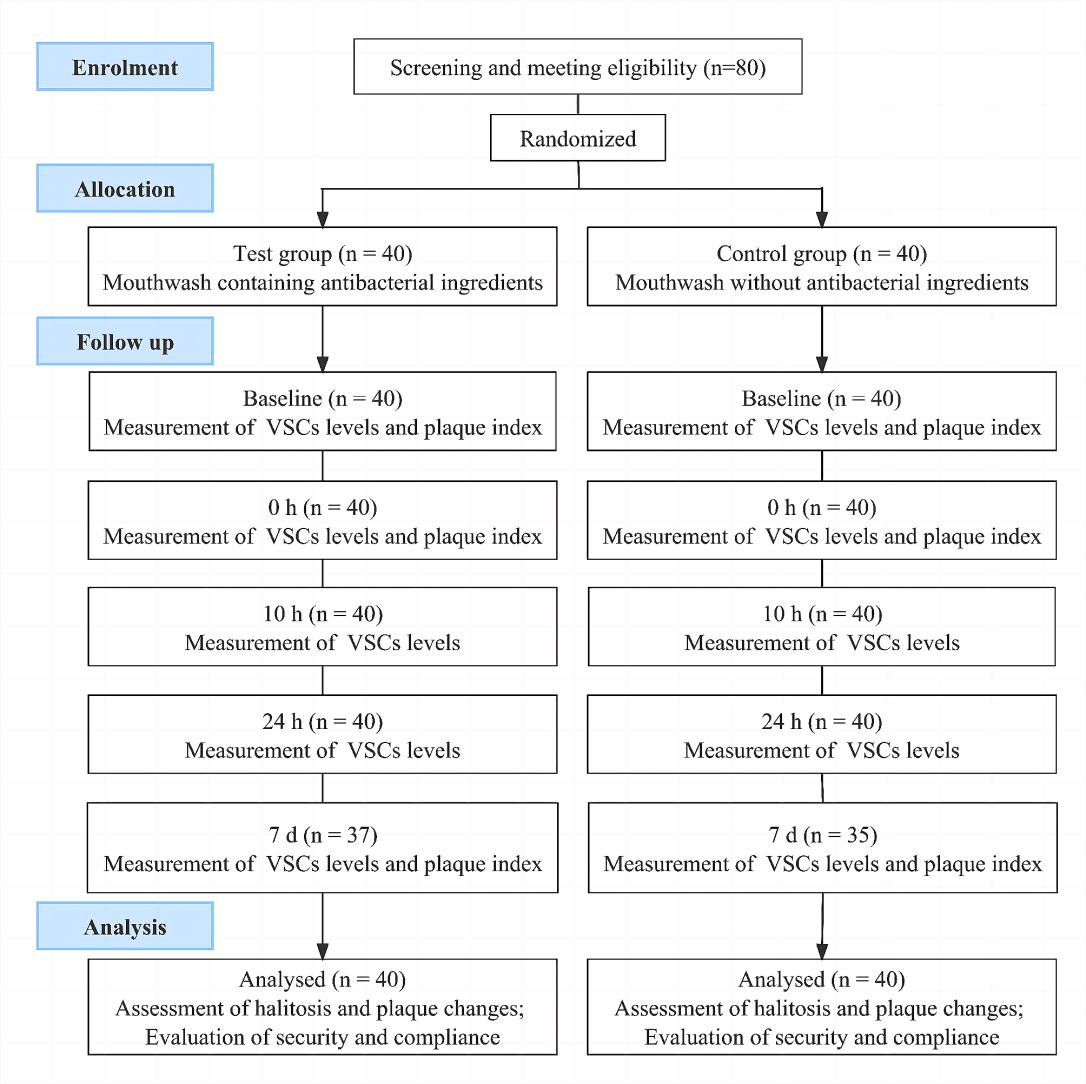
Flow chart of clinical trial

This study was approved by the Ethics committee of School of Stomatology Shandong University (Stomatological Hospital of Shandong University) (No.20,230,601). A randomized, double-blind and parallel controlled trial was registered on Chinese Clinical Trial Registry (No. ChiCTR2300073816, Date: 21/07/2023) and carried out. A total of 80 subjects who met the inclusion criteria without any exclusion criteria were recruited. They were randomly divided into the test and the control group, 40 subjects in each group. The specific operation process was shown in Fig. 1.

Inclusion criteria: (1) ages from 18 to 65 years old with good general health without serious systemic diseases; (2) having more than 20 detectable teeth with an appropriate degree of plaque or gingivitis; (3) agreeing not to use any non-test mouthwash during the trial period; (4) avoiding to consume any food producing oral odor such as garlic, leeks, stinky tofu; (5) keeping a good oral hygiene habits by brushing twice a day; (6) having VSCs levels ≥ 125 examined by the Halimeter; (7) signing an informed consent form.

Exclusion criteria: (1) concurrently joining other clinical studies; (2) using other oral health care products containing phenols or fragrances during the days of testing; (3) having the history of allergy to the tested product and its ingredients; (4) having these conditions including AIDS, insulin-dependent diabetes mellitus, anticancer chemotherapy within 6 months, immunodeficiency, autoimmune disease, or other serious medical conditions; (5) having severe gingivitis, periodontitis or oral ulcers, wearing partial or full dentures; (6) smoking or using tobacco products; (7) using antihistamines in the last week or immunosuppressants or antibiotics in the last month; (8) females with pregnancy, during breastfeeding or menstruation; (9) the presence of any disease or condition that may interfere with the examination procedure and the smooth completion of the test.

#### The usage of mouthwash

The researchers primarily explained the purpose and process of the study in detail to the recruited subjects before their informed consents were given. The researchers collected the subjects’ personal information including the medical history and dietary habits.

εAll recruited subjects underwent an oral examination followed by training on standardized brushing methods and mouthwash use. After brushing their teeth twice a day, the individuals were told to rinse their mouths with either a 20 mL BOP® mouthwash (test mouthwash) added with ε-PL, FP, and domiphen, or a non-supplemented one (control mouthwash) for 30 s, which was designated as the test group and the control group.

#### Baseline examination

Assessment of halitosis: The degree of halitosis was assessed by measuring the levels of VSCs using Halimeter [22]. Before measurement, the subject closed the mouth for 3 min. Then, the subject breathed through the nose with the mouth opening slightly, and the collector is placed 0.5 cm above the middle and posterior third of the dorsum of tongue to read the peak value on the display screen. The subjects with a mean VSCs level above 125 across the three tests were eligible for inclusion and were randomly assigned to the test and control groups.

Assessment of supragingival plaque: To determine supragingival plaque formation, six marked teeth (upper right 6, upper left 1 and 4, lower left 6 and lower right 1 and 4) were stained with the dye using cotton swab. After 1 min, each tooth was examined on four tooth surfaces, namely mesial buccal, mid-buccal, distal-buccal and lingual. Based on the PLI scoring standards [23] (Table 1), each tooth was scored as the sum of the four tooth surface scores divided by four, and the individual score was counted as the sum of the fractions of each tooth divided by the number of teeth examined.

**Table 1:** PLI scoring standards

0 = no plaque in the gingival margin area
1 = thin plaque on the tooth surface in the gingival margin area, but not visible by visual inspection. If the tooth surface is scratched with a probe, plaque can be seen
2 = medium amount of plaque can be seen on the gingival margin or adjacent surface
3 = large amount of soft scale in the gingival crease or in the gingival margin area and adjacent surface

#### **The examination of trial outcome**

The clinical trial lasted for 7 days. The VSCs levels were measured at the baseline, 0, 10, 24 h, and 7 days following the first mouthwash application, and the amount of plaque was only tested at baseline and day 7. Both the detection items and methods were identical to the baseline. In addition, the oral soft and hard tissue examination and compliance statistics were performed at 0, 10, 24 h and 7 days after the first use of mouthwash to evaluate the potential side effects of the new formula.

#### Statistical analysis

Quantitative data were described as mean, standard deviation, minimum, median and maximum. Qualitative indicators were described by frequency table, percentage or constituent ratio. Quantitative data were visualized using QQ plots, and a normal distribution compliance was assessed by the Shapiro-Wilk normality test. Homogeneity of variance was tested by Levene’s test. Quantitative data, data in line with normal distribution were compared between the two groups by *t* test, the multiple groups with equal variance were compared by ANOVA analysis, and the multiple groups with unequal variance were compared by Welch’s ANOVA test. The Wilcoxon rank-sum test was used to confirm the skewed distribution. Qualitative data were analyzed by chi-square test, Fisher exact test or Knuskal-wallis test. In addition, to evaluate the effect of the mouthwash on the breath index, a stratified analysis was performed, and severe breath was defined as those with a breath score greater than the median.

All data were analyzed using the R 15.6.0 platform. All tests were two-sided, and the confidence level was 0.05.

## Results

### The antimicrobial and bactericidal test of the combination of three ingredients

**Fig. 2:**
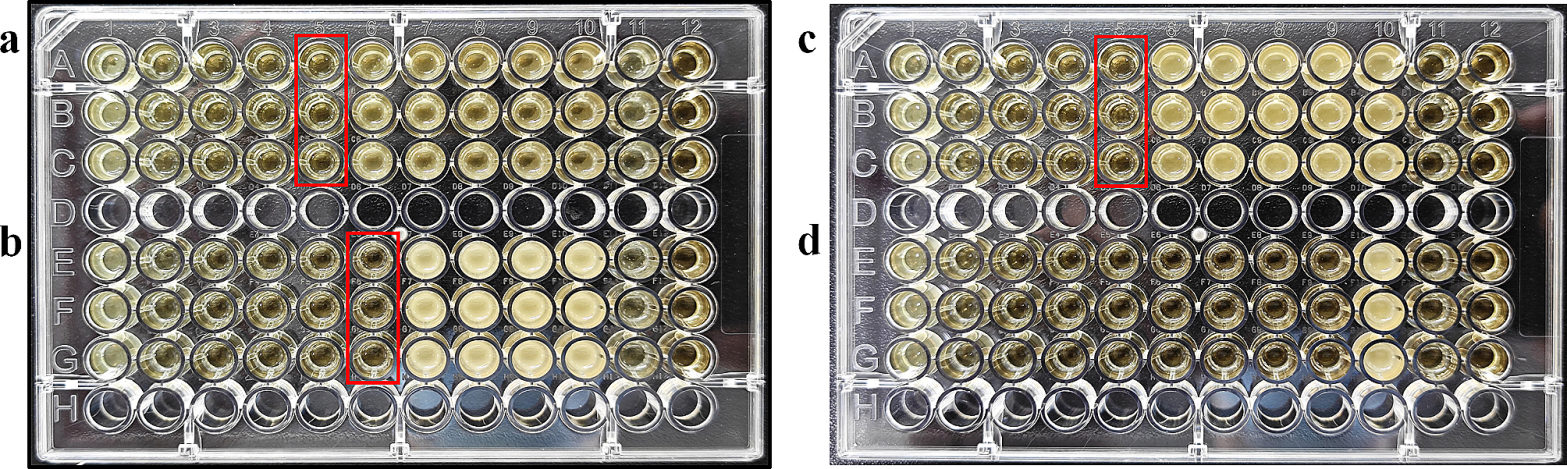
MIC of the combined solution (ε-PL, FP and domiphen) against four oral pathogens. The red boxes represent the exact MIC against the four pathogens, 1.56% (v/v) for *A. actinomycetemcomitans* (**a**), 0.78% (v/v) for *S. mutans* (**b**), 1.56% (v/v) for *F. nucleatum* (**c**), < 0.10% (v/v) for *P. gingivalis* (**d**)

**Fig. 3:**
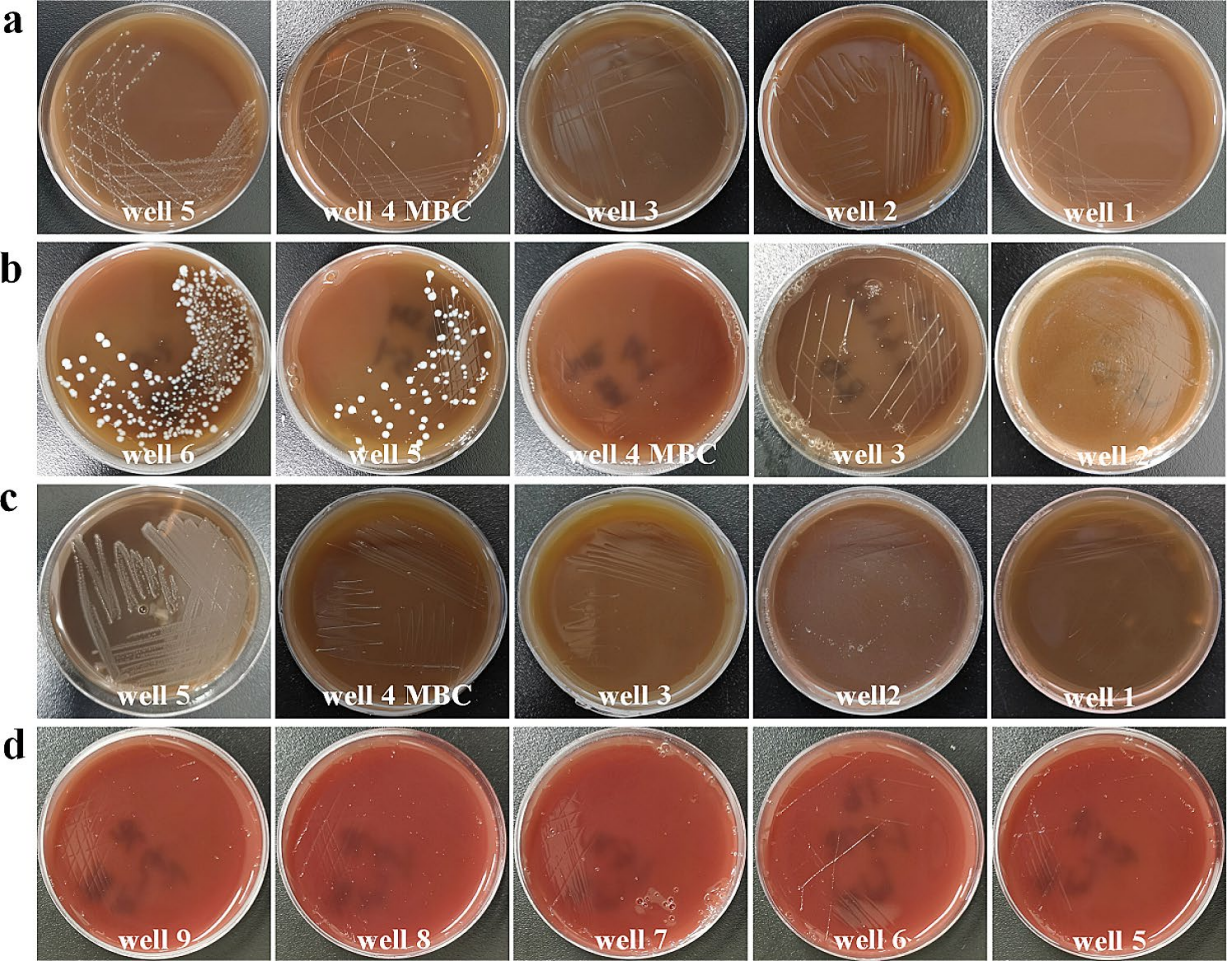
MBC of the combined solution (ε-PL, FP and domiphen) against four oral pathogens, 3.13% (v/v) for *A. actinomycetemcomitans* (**a**), *S. mutans* (**b**) and *F. nucleatum* (**c**), < 0.10% (v/v) for *P. gingivalis* (**d**)

The MIC and MBC value of the solution containing ɛ-PL, FP, and domiphen were determined by the gradient dilution method. The new mixed-antimicrobial solution showed different degrees of inhibitory and bactericidal effects against the tested bacteria as shown in Table 2; Figs. 2 and 3. The MIC and MBC values of this mixed antimicrobial solution against the tested oral pathogens were less than 1.56% (v/v) and 3.13% (v/v), respectively. *P. gingivalis* was most susceptible to the mixed antimicrobial, whereas *A. actinomycetemcomitan* and *F. nucleotum* was the least susceptible to such antimicrobial.

**Table 2:** MIC and MBC of the combined solution of ε-PL, FP and domiphen against four oral pathogens

Pathogenic bacteria	MIC (v/v%)	MBC (v/v%)
*A. actinomycetemcomitans*	1.56	3.13
*S. mutans*	0.78	3.13
*F. nucleatum*	1.56	3.13
*P. gingivalis*	< 0.10	< 0.10

### Effects of mouthwash on cell viability

**Fig. 4:**
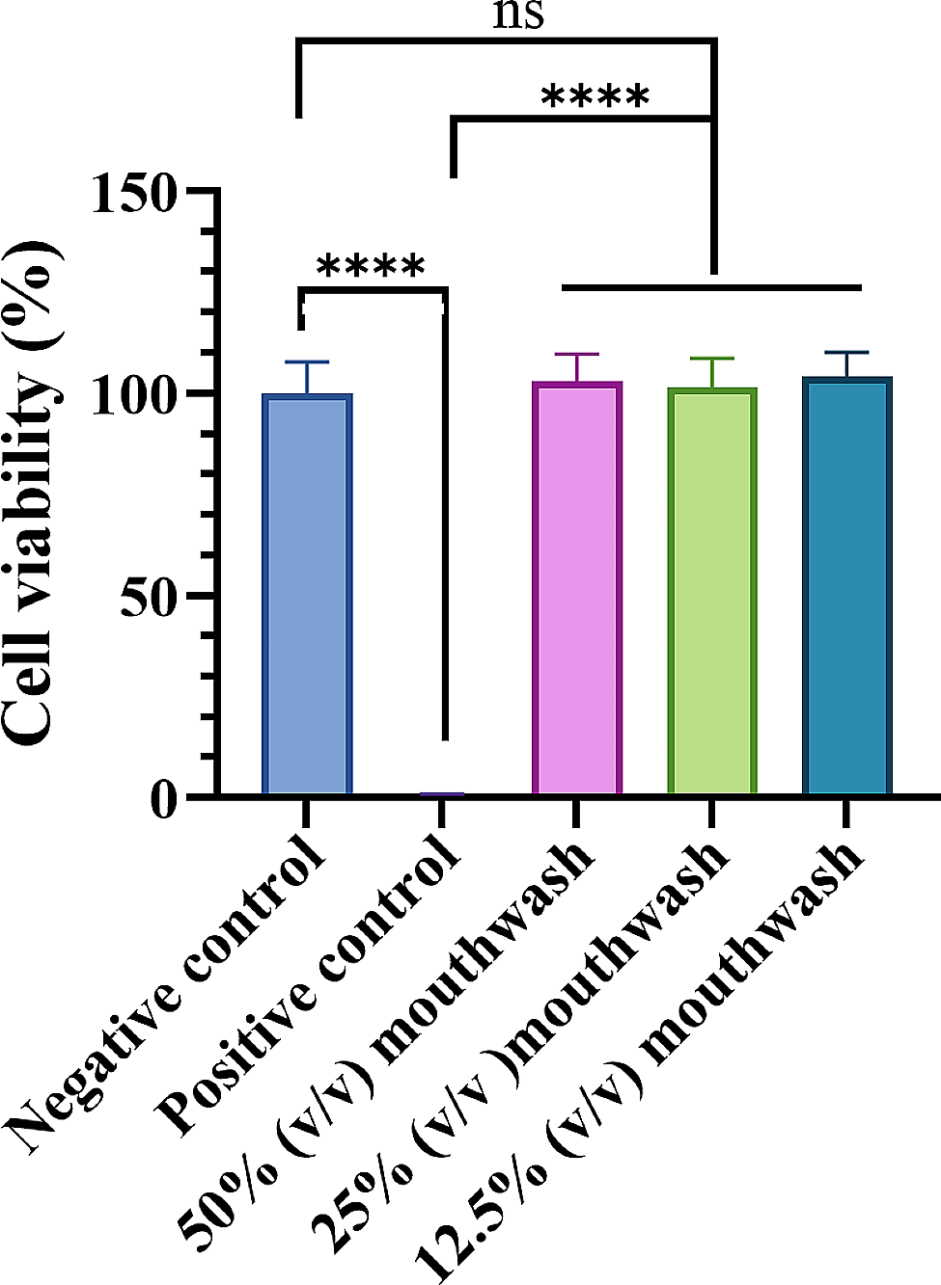
The cytotoxicity of different concentrations of mouthwash on HGFs. ^****^P < 0.0001, ns: nonsignificant

The CCK-8 assay (Fig. 4) was used to assess the effect of the mouthwash on HGF viability. After exposure for 30 s, the positive control group showed great cytotoxicity to the HGFs, and the average survival rate was only 0.68 ± 0.22%, while the cell viability of the negative control group and the test group was more than 100%, which was significantly different from that of the positive control group (*P* < 0.0001).

### Basic information of subjects included in clinical trials

Herein, a mouthwash containingthree anti-microbial ingredients was formulated and conducted a randomized, double-blind, parallel, and controlled clinical trial (Fig. 1). The clinical trial enrolled a total of 80 participants, comprising 47 females and 33 males, with ages ranging from 18 to 61 years and a mean age of 39 years. Before the trial, VSCs and PLI levels were measured for all subjects (Table S1). The participants were equally and randomly assigned to either the test or control groups, with 40 subjects in each. Analysis depicted in Table 3 revealed no significant disparity in gender, age, VSCs levels, or PLI between the test and control groups (*P* > 0.1).

**Table 3:** Basic information of subjects in the test and control group

Index		Test group	Control group	*P* value
gender	female	25(62.5%)	22(55.0%)	> 0.1
	male	15(37.5%)	18(45.0%)	> 0.1
age (years)	mean ± SD	38 ± 11.9	40 ± 11.7	> 0.1
	median	38	37.5	> 0.1
	interval	19–59	18–61	NA
VSCs levels	mean ± SD	346.3 ± 103.0	331.1 ± 90.5	> 0.1
	median	332	359	> 0.1
	interval	141–549	142–521	NA
PLI	mean ± SD	1.87 ± 0.77	1.91 ± 0.58	> 0.1
	median	2	2	> 0.1
	interval	1–3	1–3	> 0.1

### The test mouthwash improved halitosis status

The VSCs levelwas tested and analyzed at the baseline, and 0 h, 10 h, 24 h, and 7 days of mouthwash use. The Shapiro-Wilk normal distribution test indicated a normal distribution of VSCs levels in both groups and at each time point (Fig. S1). Further, the Levene’s Test for homogeneity of variance demonstrated homogeneous variances of VSCs levels in both the test and control groups at each time point (Table S2) (*P* ≥ 0.05).

**Fig. 5:**
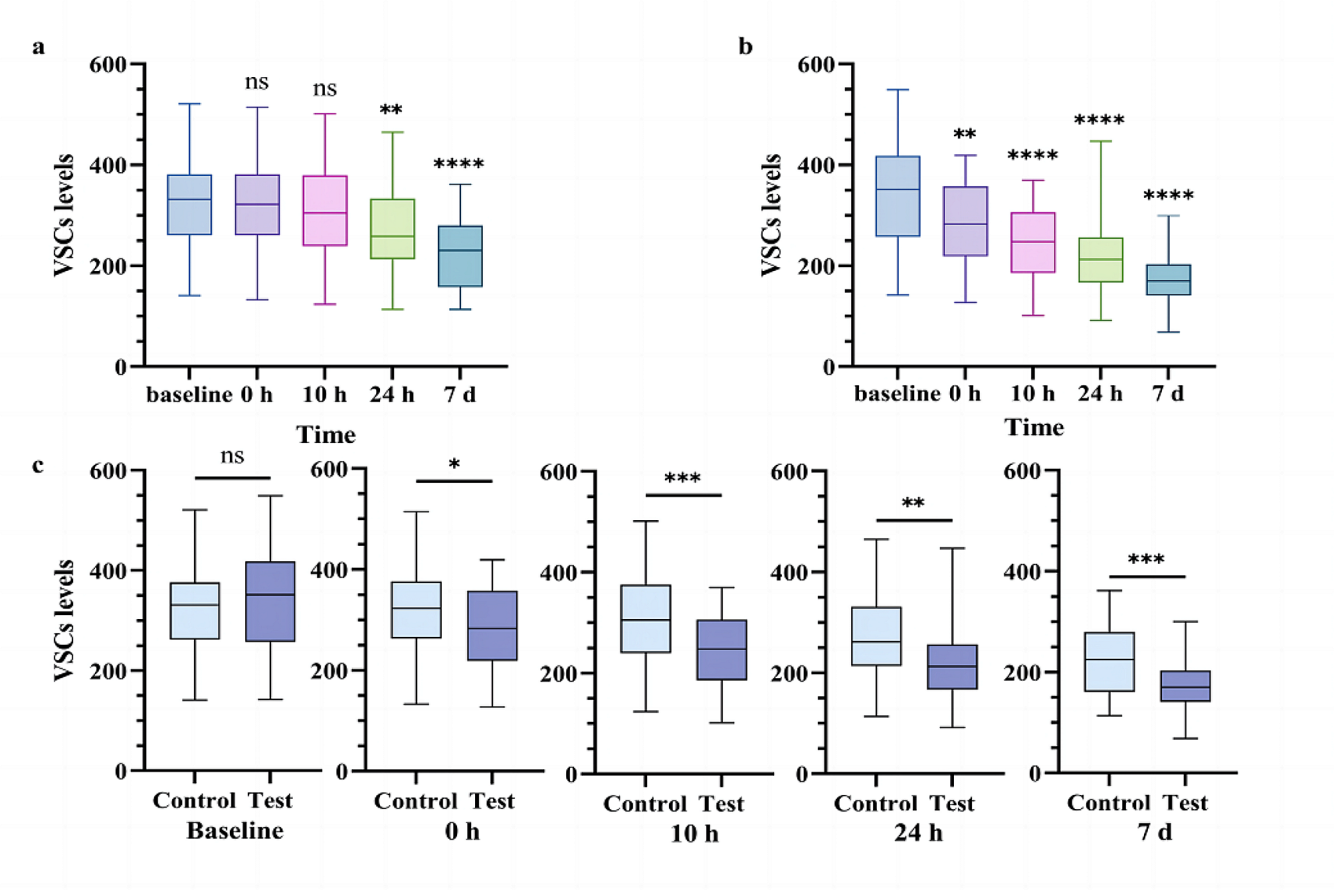
Comparison of the VSCs levels among two groups at 5 time points. **a** Changes of VSCs levels at 5 time points in control group. **b** Changes of VSCs levels at 5 time points in test group. **c** Comparison of the VSCs levels between test and control group at 5 time points. ^*^P < 0.05, ^**^P < 0.01, ^***^P < 0.001, ^****^P < 0.0001, ns: nonsignificant

The results showed that a huge reduction of VSC levels was observed in the test group (rinsing with mouthwash supplemented with mixed antibacterial), compared with the control group (rinsing with mouthwash without supplement). After 7 days, the VSCs levels decreased by 50.3% and 32.1% in the test and control groups respectively, compared with their baseline (*P* < 0.01) (Fig. 5a, b). Moreover, at any duration of mouthwash use (0, 10, 24 h, 7days), the levels of VSCs of the test group were lower than those of control group (Fig. 5c, Table S3).

### The test mouthwash improved “severe halitosis” condition

**Fig. 6:**
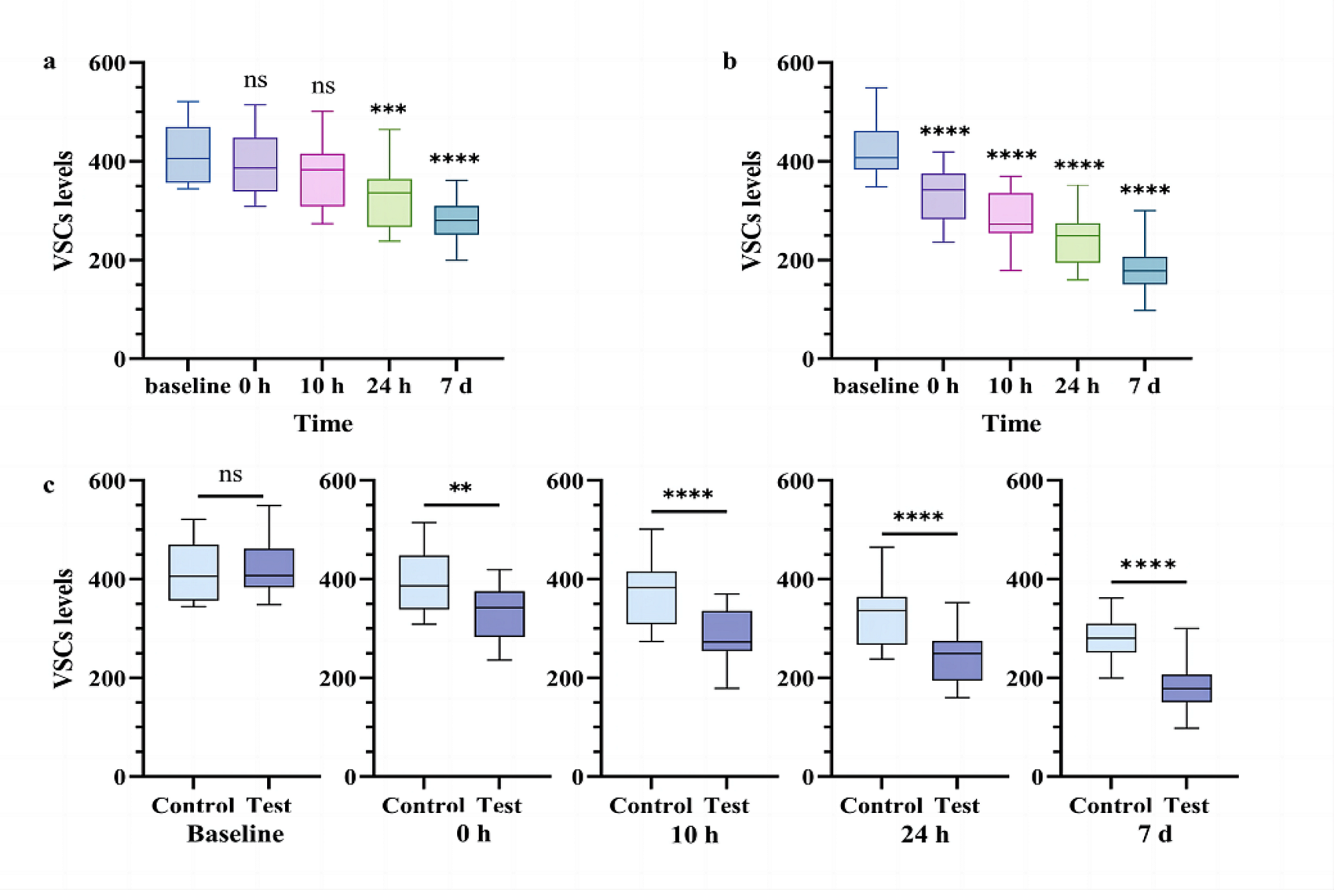
Effect of without/with combined antibacterials on subjects with “severe halitosis”. **a** Changes of VSCs levels at 5 time points within the “severe halitosis” subjects of control group. **b** Changes of VSCs levels at 5 time points in the “severe halitosis” subjects of test group. **c** Comparison of VSCs levels between “severe halitosis” subjects of the two groups at 5 time points. ^**^P < 0.01, ^***^P < 0.001, ^****^P < 0.0001, ns: nonsignificant

At the baseline, 23 out of 40 individuals in the test group and 17 out of 40 in the control group exhibited “severe halitosis” (VSCs levels above the median), and there was no statistically significant difference in VSCs levels between the two groups. Building upon this observation, we investigated the efficacy of mouthwash in addressing “severe halitosis”. The findings highlighted a more pronounced improvement in VSCs levels among the subjects with “severe halitosis” in the test group. Following 7 days of mouthwash use, the average VSCs levels in the control group decreased from 413.6 to 282.2, reflecting a 31.77% decrease. In contrast, the average VSCs levels in the test group dropped from 420.1 to 180.5, showcasing a decrease rate of 57.03% (Fig. 6a, b and Table S4) and surpassing the overall VSCs levels decrease (50.27%). Additionally, the inter-group disparity in VSCs levels escalated with prolonged mouthwash use, transitioning from − 6.5 at baseline to 101.7 after 7 days (Fig. 6c and Table S4).

### The test mouthwash reduced supragingival plaque

**Fig. 7:**
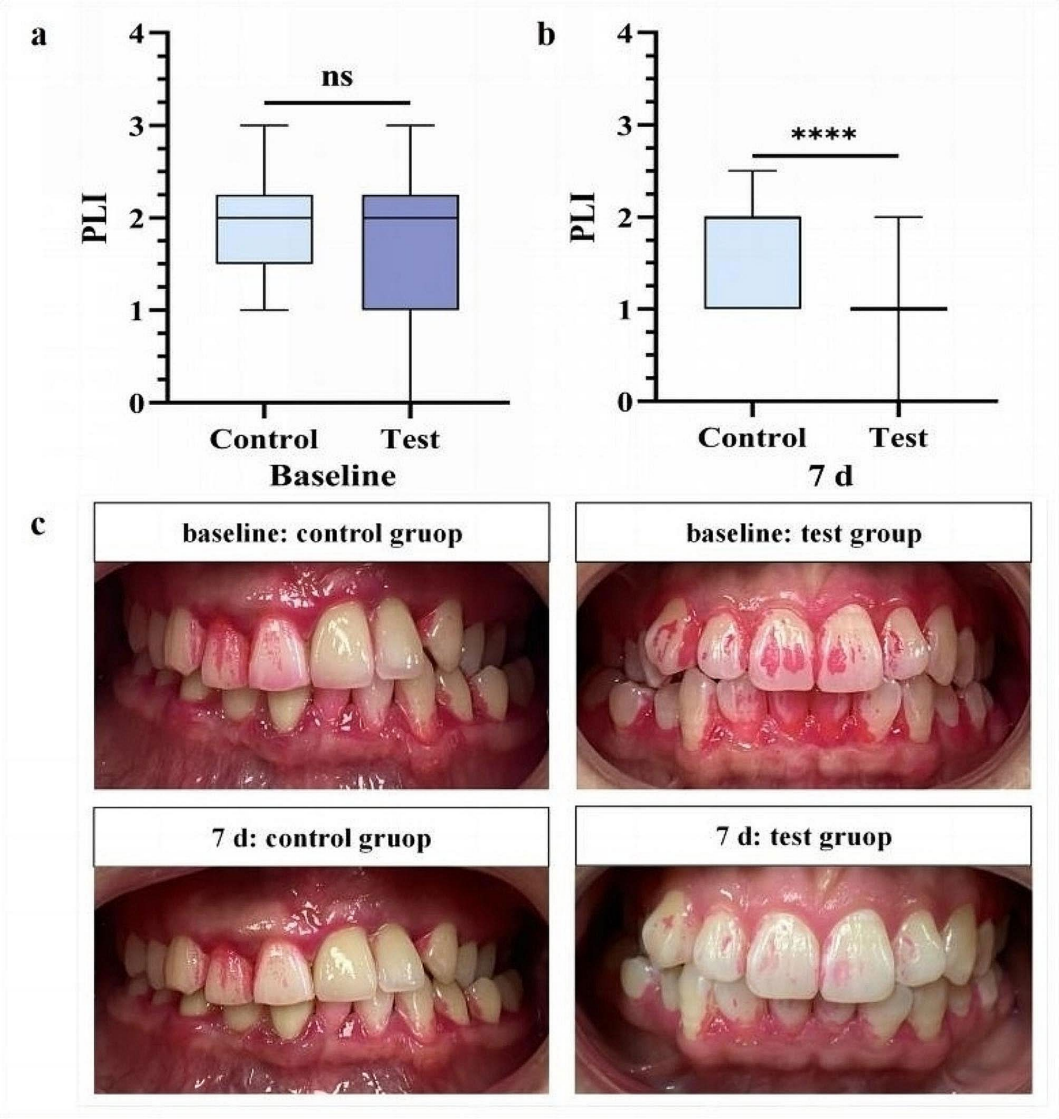
Plaque removal effect of mouthwash supplemented with combined antibacterials. **a** PLI comparison between test and control groups at baseline. **b** PLI comparison between test and control groups after 7 days. **c** Representative pictures of supragingival plaque changes in the test and control group. ^****^P < 0.0001, ns: nonsignificant

Plaque index (PLI) was scored in both control and test groups after using mouthwash without and with antibacterials as depicted in Fig. 7 and Table S5. At baseline, there was no significant difference of PLI between the test and control groups (*P* > 0.1) (Fig. 7a, Table S5). At 7 days of mouthwash use, PLI of the test and control groups was decreased by approximately 54.6% and 8.4%, respectively (*P* < 0.01) (Fig. 7b, Table S5), which could observed the difference by the naked eye (Fig. 7c).

### Neither mouthwash caused adverse reactions in the subjects

During the whole trial, we always paid attention to the oral health status of the subjects and perform security checks during the return visit, and the compliance statistics were carried out after the trial. No subjects reported adverse oral soft and hard tissue reactions related to mouthwash during the trial, including but not limited to oral burning sensation, taste changes, tooth sensitivity changes, and pathological changes as shown in Table 4.

**Table 4:** Examination of oral soft and hard tissues of the subjects

Index	Test group	Control group	*P* value
Oral soft and hard tissue examination			
abnormal	0(0%)	0(0%)	NA
normal	37(100%)	35(100%)	NA

The compliance statistics showed that 3 of the 40 subjects in the test group were lost to follow-up on the 7th day. The test mouthwash should be used 560 times, and the actual use was 560 times, and the compliance rate was 100%. 5 of 40 subjects in the control group were lost to follow-up on the 7th day. The control mouthwash should be used 560 times and was actually used 504 times, with a compliance rate of 90%. The compliance of the two groups was acceptable, and the compliance of the test group was higher than that of the control group.

## Discussion

Oral health is not only related to mouth, tooth and orofacial structures to perform functions effectively and properly but also directly and indirectly affects general health. Mouthwash has been recommended to be the essential for cleaning teeth and gum and freshening the breath. At present, commercial mouthwashes commonly contain some active ingredients including chlorhexidine, cetylpyridonium chloride, fluoride, and alcohol that act as antibacterial substances, however some users may encounter with cytotoxicity and various side effects caused by such active ingredients [24]. In this study, the newly developed antibacterial combination of domiphen and other two non-toxic AMPs namely ɛ-PL and FP was investigated for its inhibitory and bactericidal effects against some oral pathogens, and the mouthwash supplemented with this antibacterial combination in reducing effects of halitosis and supragingival plaque formation was validated by a clinical trial.

The antimicrobial effects and safety of the efficacy ingredients used in this study have been evaluated in several studies. Domiphen can inhibit the growth of *Acinetobacter baumannii*, *Staphylococcus aureus*, *Escherichia coli*, *Candida albic*ans, *Aspergillus fumigatus* and other pathogens at low concentrations [25, 26]. As a quaternary ammonium compound, domiphen also has some advantages such as low toxicity and chemical stability [27]. ε-PL shows a potent antibacterial efficacy against both gram positive and negative bacteria [28], as well as oral aerobic groups [29]. α. The antimicrobial properties of ε-PL have been well established in the food industry and it is increasingly used in oral applications, including dental adhesives, implant surface modification, and antibacterial agents [30, 31]. FP is a recombinant antimicrobial peptide derived from BMAP-27. Compared with the parental AMPs, FP has the advantages of lower molecular weight, higher antibacterial and lower hemolytic activity. Study showed that FP had an MIC of 125 µg/mL against *Cronobacter sakazakii* and completely cleared *Cronobacter sakazakii* biofilms at 2 × MIC, also FP showed extremely low cytotoxicity on human erythrocytes [21].

The combined antimicrobials with synergistic interaction are frequently proposed to enhance their antimicrobial properties and to reduce drug resistant strains [32]. For example, the co-application of domiphen and miconazole reduced the number of viable *Candida albicans* cells in biofilm by 1000 folds, and increased its therapeutic activity against *Vulvovaginal candidiasis* in rat model [33]. ε-PL and nisin A have synergistic antibacterial effects on gram positive foodborne pathogens such as *Bacillus cereus* and *Listeria monocytogenes* [34]. Additionally, ε-PL coupled with erythromycin demonstrated relatively strong antibacterial activity against *Staphylococcus aureus* and *Enterococcus faecalis*, compared with the ε-PL alone [35]. In this study, an innovative combination of ε-PL, FP and domiphen was developed and its superior antibacterial effect was proved. We speculate that the excellent antibacterial effect is attributed to the synergistic effect of the three ingredients, and this study also provides a new member for the antibacterial components of oral care products.

Current studies have shown that *P. gingivalis* and *F. nucleatum* are the main pathogens for chronic periodontitis, *A. actinomycetemcomitans* is the main pathogen of aggressive periodontitis, and *S. mutans* is the main pathogen of dental caries. *P. gingivalis* can adhere to and invade the host gingiva, destroy the periodontal tissue by using virulence factors and metabolites, and induce the inflammatory response, thereby promoting the occurrence of periodontitis and even systemic diseases [36]. *F. nucleatum* adheres to periodontal tissues by surface proteins and promotes the formation of dental plaque biofilm and the occurrence of periodontitis by copolymerization with other pathogenic bacteria [37]. *A. actinomycetemcomitans* can synthesize and secrete a variety of virulence factors to facilitate its better colonization in the host body, and accelerate the process of periodontitis by affecting the host’s immune regulation ability and destroying the periodontal tissue [38]. *S. mutans* produces organic acids by decomposing dietary carbohydrates, which leads to the acidification of dental plaque and enamel demineralization and promotes the occurrence of dental caries [39]. The antibacterial test results of present study showed that lower concentrations of the combination of three components could effectively inhibit the proliferation of these four oral pathogens. Due to the critical role of these pathogenic bacteria in periodontitis and dental caries, we suggested that the antibacterial mouthwash developed in this study may have potential application in preventing the occurrence of oral diseases.

Dental plaque is a soft microbial biofilm deposited on the tooth surfaces mainly causing dental caries and periodontal diseases [40]. In this clinical trial, after rinsing with the test mouthwash supplemented with ε-PL, FP and domiphen for 7 days, the quantity of supragingival plaque was reduced by 54.55%, compared with those of chlorhexidine containing mouthwash (66.26%) [41]. It showed that the newly developed mouthwash demonstrated slightly weaker anti-dental plaque property than the chlorhexidine containing one. However, the test mouthwash ε showed stronger anti-dental plaque activity than those of propolis or hydrogen peroxide containing rinse (reduced by 50.00%) [41]. Moreover, the mouthwash newly developed here showed much higher anti-dental plaque activity than the one containing probiotics (reduced only 27.65%, after rinsing for 30 days) [42]. Therefore, the mouthwash supplemented with ε-PL, FP and domiphen may be considered as an excellent anti-dental plaque rinse.

An overgrowth of anaerobic bacteria in the mouth can produce VSCs, which is the direct cause of halitosis [43]. In this study, the VSC levels in the groups of control, test, and test with “severe halitosis” were reduced by 32.12%, 50.27%, and 57.03%, respectively. In a previous study, Sharma et al. [44] used spectrophotometry to compare the efficacy of chlorhexidine, hydrogen peroxide, and Tulsi extract mouthwash in reducing halitosis, and the VSCs reduction rates after 14 days were 48.43%, 46.10%, and 18.59%, respectively, which were lower than test mouthwash of this study. The difference also demonstrated the potential anti-halitosis ability of the mouthwash supplemented with ε-PL, FP and domiphen.

In this study, we also evaluated the safety of the mouthwash using the CCK-8 assay and clinical tests. Results showed that the mouthwash did not significantly inhibit the viability of HGFs at the test concentration. During the clinical trial, no participant reported any oral soft or hard tissue side effects related to the mouthwash. Therefore, it is believed that this mouthwash is a safe and effective oral care product.

The main limitation of this study is the lack of long-term follow-up data to verify the efficacy of this newly developed mouthwash in reducing halitosis and dental plaque, and the generalizability is limited because the specific populations such as smokers and patients with oral diseases were excluded. Future studies will conduct long-term follow-up in more heterogeneous populations to obtain more practical results. In addition, the effects of mouthwash on the inhibition of drug-resistant oral pathogens and on the overall oral microbiota need to be investigated.

## Conclusions

The combination of ε-PL, FP and domiphen could effectively inhibited the proliferation of oral pathogens *P. gingivalis*, *F. nucleatum*, *S. mutans* and *A. actinomycetemcomitans* at low concentrations, showing excellent antibacterial efficacy. The newly developed mouthwash supplemented with the three ingredients also demonstrated anti-plaque formation and anti-halitosis properties in a clinical trial study. This study laid an inspiration for the development and application of antibacterial ingredients including ε-PL, FP and domiphen in the oral care products.

### Electronic supplementary material

Below is the link to the electronic supplementary material.

Supplementary Material 1

## Data Availability

All data generated or analysed during this study are included in this published article and its supplementary information files.

## Abbreviations

AMPs: Antimicrobial peptides
BHI: Brain Heart Infusion
ε-PL: ε-poly-L-lysine
FP: funme peptide
HGFs: Human gingival fibroblasts
MIC: Minimum Inhibitory Concentration
MBC: Minimum Bactericidal Concentration
PLI: Plaque Index
VSCs: volatile sulfur compounds
