# Supplementary material for: Efficacy of a mouthwash containing ε-poly-L-lysine, funme peptides and domiphen in reducing halitosis and supragingival plaque: a randomized clinical trial
Source: BMC Oral Health. 2024 May 3;24:525. doi: 10.1186/s12903-024-04255-0 (PMC11069150; doi:10.1186/s12903-024-04255-0)
Supplement: Supplementary file 1 — Supplementary Material 1 [file 12903_2024_4255_MOESM1_ESM.docx]

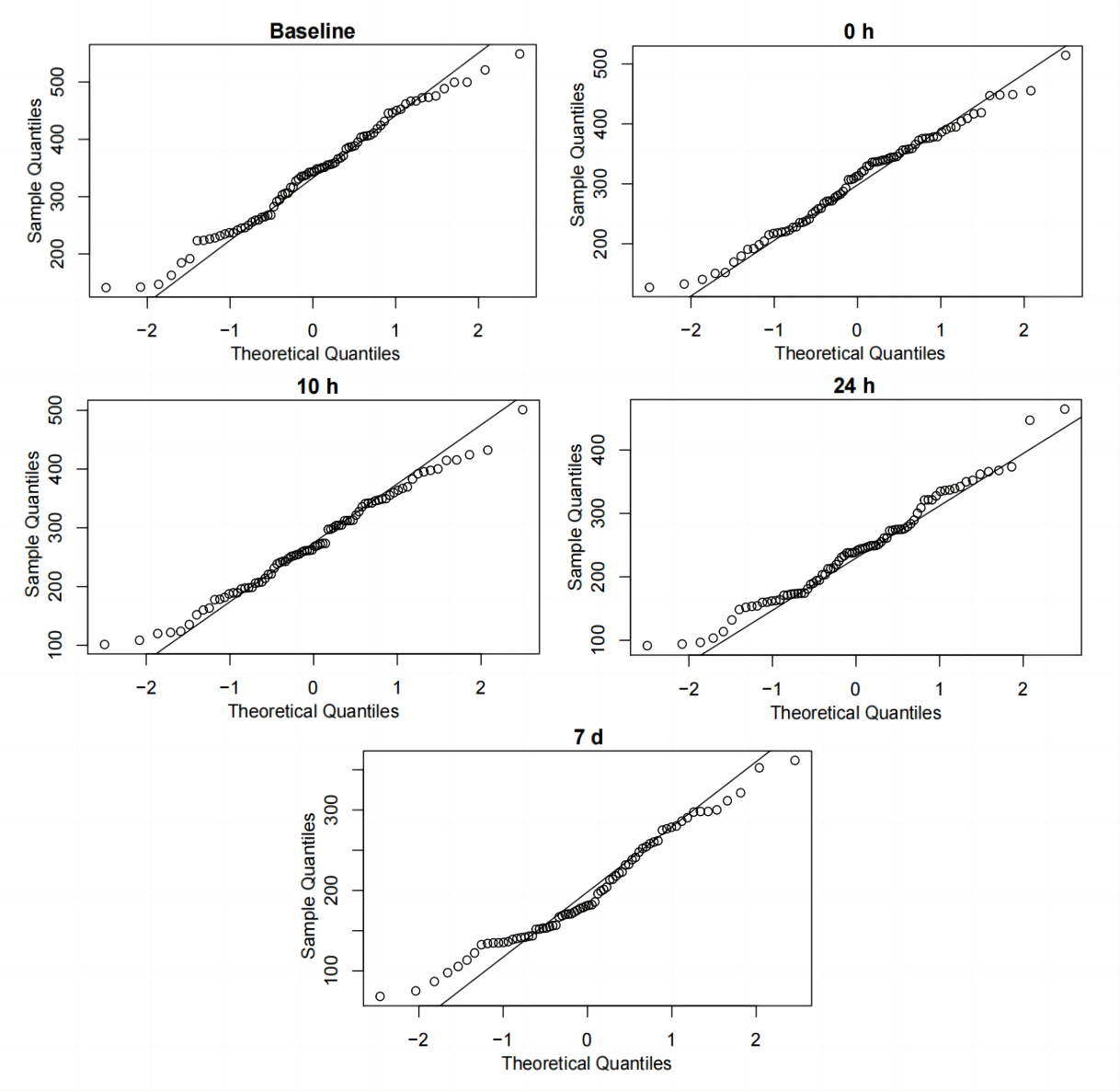


**Fig. S1** QQ plot of VSCs levels of subjects.

**Table S1.** Basic information of the subjects

| **ID** | **Group** | **Gender** | **Age** | **Level of VSCs** | **PLI** |
| --- | --- | --- | --- | --- | --- |
| BOPqx1 | Control | F | 39 | 327.2633333 | 1 |
| BOPqx2 | Control | F | 50 | 466.9833333 | 3 |
| BOPqx3 | Control | F | 59 | 473.1533333 | 2.2 |
| BOPqx4 | Control | M | 47 | 452.6266667 | 1 |
| BOPqx5 | Control | M | 38 | 354.9233333 | 2.25 |
| BOPqx6 | Control | F | 32 | 357.1433333 | 2.5 |
| BOPqx7 | Control | M | 41 | 305.7633333 | 1 |
| BOPqx8 | Control | F | 36 | 291.2933333 | 2 |
| BOPqx9 | Control | F | 32 | 237.1433333 | 1 |
| BOPqx10 | Control | M | 31 | 365.9333333 | 1 |
| BOPqx11 | Control | M | 37 | 258.7466667 | 2.5 |
| BOPqx12 | Control | M | 20 | 141.1833333 | 2 |
| BOPqx13 | Control | M | 51 | 245.05 | 2 |
| BOPqx14 | Control | F | 37 | 306.0933333 | 1 |
| BOPqx15 | Control | F | 50 | 335.9933333 | 2 |
| BOPqx16 | Control | M | 61 | 472.49 | 1.5 |
| BOPqx17 | Control | F | 33 | 184.59 | 1 |
| BOPqx18 | Control | F | 24 | 226.1633333 | 2 |
| BOPqx19 | Control | F | 51 | 241.5966667 | 1.5 |
| BOPqx20 | Control | M | 53 | 344.3133333 | 2 |
| BOPqx21 | Control | M | 36 | 350.46 | 2.5 |
| BOPqx22 | Control | F | 30 | 264.8633333 | 2.5 |
| BOPqx23 | Control | F | 52 | 405.92 | 1.5 |
| BOPqx24 | Control | F | 53 | 386.28 | 1.5 |
| BOPqx25 | Control | F | 52 | 418.34 | 1.5 |
| BOPqx26 | Control | F | 56 | 356.0266667 | 2 |
| BOPqx27 | Control | M | 26 | 267.77 | 1.5 |
| BOPqx28 | Control | M | 44 | 337.81 | 2 |
| BOPqx29 | Control | F | 49 | 237.0233333 | 2.5 |
| BOPqx30 | Control | M | 37 | 303.2466667 | 2.25 |
| BOPqx31 | Control | M | 32 | 343.1166667 | 2 |
| BOPqx32 | Control | F | 26 | 445.6533333 | 2 |
| BOPqx33 | Control | F | 45 | 359.5066667 | 2.25 |
| BOPqx34 | Control | M | 30 | 191.73 | 1 |
| BOPqx35 | Control | M | 18 | 282.5133333 | 2 |
| BOPqx36 | Control | F | 27 | 250.16 | 2 |
| BOPqx37 | Control | M | 58 | 499.51 | 3 |
| BOPqx38 | Control | F | 27 | 316.48 | 1 |
| BOPqx39 | Control | M | 52 | 316.0966667 | 2.5 |
| BOPqx40 | Control | F | 28 | 521.1133333 | 2 |
| BOPqx41 | Test | F | 53 | 342.2733333 | 2.5 |
| BOPqx42 | Test | F | 55 | 367.5133333 | 2 |
| BOPqx43 | Test | F | 37 | 263.9 | 1 |
| BOPqx44 | Test | M | 44 | 142.18 | 2.5 |
| BOPqx45 | Test | F | 25 | 330.76 | 3 |
| BOPqx46 | Test | M | 41 | 466.6733333 | 2.25 |
| BOPqx47 | Test | F | 38 | 348.4233333 | 2.25 |
| BOPqx48 | Test | M | 21 | 351.2433333 | 0 |
| BOPqx49 | Test | F | 47 | 411.1733333 | 3 |
| BOPqx50 | Test | M | 59 | 446.6766667 | 2 |
| BOPqx51 | Test | F | 47 | 488.5333333 | 2 |
| BOPqx52 | Test | F | 52 | 405.3366667 | 1 |
| BOPqx53 | Test | F | 28 | 255.6066667 | 1.5 |
| BOPqx54 | Test | F | 19 | 335.16 | 2 |
| BOPqx55 | Test | M | 52 | 549.0666667 | 2 |
| BOPqx56 | Test | M | 31 | 245.8 | 2 |
| BOPqx57 | Test | F | 49 | 403.49 | 2 |
| BOPqx58 | Test | F | 22 | 146.98 | 1 |
| BOPqx59 | Test | M | 57 | 499.7333333 | 2 |
| BOPqx60 | Test | F | 55 | 387.15 | 2 |
| BOPqx61 | Test | F | 27 | 231.5366667 | 3 |
| BOPqx62 | Test | F | 26 | 227.9766667 | 1 |
| BOPqx63 | Test | M | 47 | 348.4133333 | 2 |
| BOPqx64 | Test | M | 35 | 223.7733333 | 1 |
| BOPqx65 | Test | F | 22 | 162.7433333 | 1 |
| BOPqx66 | Test | F | 29 | 475.7566667 | 2 |
| BOPqx67 | Test | F | 28 | 267.8466667 | 3 |
| BOPqx68 | Test | M | 29 | 294.4833333 | 2 |
| BOPqx69 | Test | F | 47 | 450.4233333 | 1 |
| BOPqx70 | Test | M | 30 | 395.0233333 | 1 |
| BOPqx71 | Test | F | 47 | 424.34 | 3 |
| BOPqx72 | Test | F | 27 | 259.44 | 3 |
| BOPqx73 | Test | M | 51 | 371.4 | 1 |
| BOPqx74 | Test | F | 38 | 388.7366667 | 2 |
| BOPqx75 | Test | M | 42 | 383.4133333 | 3 |
| BOPqx76 | Test | M | 27 | 461.6833333 | 2 |
| BOPqx77 | Test | F | 22 | 234.6666667 | 1 |
| BOPqx78 | Test | F | 41 | 407.27 | 1 |
| BOPqx79 | Test | F | 45 | 431.4366667 | 1.5 |
| BOPqx80 | Test | M | 28 | 223.1333333 | 1.5 |

**Table S2.** Results of homogeneity of variance test at each time point between test and control groups

| **Time point** | **Levene's Test** |
| --- | --- |
| baseline | F value=1.0738, *P*=0.3033 |
| 0 h | F value=0.3999, *P*=0.5290 |
| 10 h | F value=0.4864, *P*=0.4876 |
| 24 h | F value=0.3602, *P*=0.5501 |
| 7 d | F value=3.9992, *P*=0.0494 |

**Table S3.** Comparison of VSCs level of test and control groups

| Time points | Test group（n=40） | Control group（n=40） | *P* value |
| --- | --- | --- | --- |
| baseline | 346.3±103.0 | 331.1±90.5 | 0.485 |
| 0 h | 284.0±83.3 | 322.6±83.8 | 0.042 |
| 10 h | 240.9±76.1 | 306.8±85.4 | 0.0004 |
| 24 h | 215.96±74.3 | 266.1±78.3 | 0.004 |
| 7 d | 172.2±56.5 (n=37) | 224.6±69.0 (n=35) | 0.001 |
| *P* value | < 0.01 | < 0.05 |  |

**Table S4.** Comparison of VSCs level in subjects with severe halitosis

| Time points | Test group（n=23） | Control group（n=17） | *P* value |
| --- | --- | --- | --- |
| baseline | 420.1±52.8 | 413.6±59.2 | 0.7136 |
| 0 h | 333.8±48.1 | 389.0±59.7 | 0.0025 |
| 10 h | 282.4±54.0 | 369.6±61.1 | 2.682e-05 |
| 24 h | 237.1±50.5 | 322.9±59.6 | 1.69e-05 |
| 7 d | 180.5±49.3 (n=13) | 282.2±45.0 (n=22) | 7.344e-07 |
| *P* value | < 0.01 | < 0.05 |  |

**Table S5.** Comparison of PLI between test and control groups in different time point

| Time points | Test group | Control group | *P* value |
| --- | --- | --- | --- |
| baseline | 1.87±0.77 (n=40) | 1.91±0.58 (n=40) | > 0.1 |
| 7 d | 0.85±0.56 (n=37) | 1.75±0.53 (n=35) | < 0.01 |
| *P* value | < 0.01 | > 0.1 |  |
